# PK-sensitive PrP^Sc^ Is Infectious and Shares Basic Structural Features with PK-resistant PrP^Sc^

**DOI:** 10.1371/journal.ppat.1002547

**Published:** 2012-03-01

**Authors:** Gustavo Sajnani, Christopher J. Silva, Adriana Ramos, Miguel A. Pastrana, Bruce C. Onisko, Melissa L. Erickson, Elizabeth M. Antaki, Irina Dynin, Ester Vázquez-Fernández, Christina J. Sigurdson, J. Mark Carter, Jesús R. Requena

**Affiliations:** 1 Department of Medicine, School of Medicine, University of Santiago de Compostela, Santiago de Compostela, Galiza, Spain; 2 Western Regional Research Center, United States Department of Agriculture, Albany, California, United States of America; 3 Department of Pathology, University of California, Davis, Davis, California, United States of America; 4 Department of Pathology, University of California, San Diego, La Jolla, California, United States of America; Dartmouth Medical School, United States of America

## Abstract

One of the main characteristics of the transmissible isoform of the prion protein (PrP^Sc^) is its partial resistance to proteinase K (PK) digestion. Diagnosis of prion disease typically relies upon immunodetection of PK-digested PrP^Sc^ following Western blot or ELISA. More recently, researchers determined that there is a sizeable fraction of PrP^Sc^ that is sensitive to PK hydrolysis (sPrP^Sc^). Our group has previously reported a method to isolate this fraction by centrifugation and showed that it has protein misfolding cyclic amplification (PMCA) converting activity. We compared the infectivity of the sPrP^Sc^ versus the PK-resistant (rPrP^Sc^) fractions of PrP^Sc^ and analyzed the biochemical characteristics of these fractions under conditions of limited proteolysis. Our results show that sPrP^Sc^ and rPrP^Sc^ fractions have comparable degrees of infectivity and that although they contain different sized multimers, these multimers share similar structural properties. Furthermore, the PK-sensitive fractions of two hamster strains, 263K and Drowsy (Dy), showed strain-dependent differences in the ratios of the sPrP^Sc^ to the rPrP^Sc^ forms of PrP^Sc^. Although the sPrP^Sc^ and rPrP^Sc^ fractions have different resistance to PK-digestion, and have previously been shown to sediment differently, and have a different distribution of multimers, they share a common structure and phenotype.

## Author Summary

Prion diseases are protein misfolding disorders. Different strains of prions are known to have variable resistance to proteinase K (PK) digestion. Furthermore, the same strain possesses both a PK sensitive (sPrP^Sc^) and PK resistant (rPrP^Sc^) aggregate of PrP. We developed methods to isolate the sPrP^Sc^ from rPrP^Sc^ fraction of the 263K strain of hamster-adapted scrapie. Both fractions were infectious, but have different physico-chemical properties. When we analyzed the lesion targets in the brain produced by each fraction they were essentially identical, suggesting that they were the same strain. The biochemical differences in the phenotypes of these two fractions are due to different sized multimers that share common structural properties. Furthermore, the comparison of the sensitive fractions of two hamster strains, 263K and Drowsy (Dy), showed strain-dependent differences in the ratios of the PK-sensitive to the PK-resistant forms of PrP^Sc^.

## Introduction

The prion (PrP^Sc^) is the infectious agent responsible for a suite of different rare animal and human diseases known as transmissible spongiform encephalopathies (TSEs) [1], [2], [3], [4], [5]. PrP^Sc^ is able to convert a normal cellular prion protein (PrP^C^) into PrP^Sc^ when both isoforms make contact, and thereby propagate an infection. The conversion of PrP^C^ into PrP^Sc^ involves a conformational change of the protein in which the total amount of β-sheet increases and that of α-helical secondary structure decreases or perhaps disappears [6], [7]. PrP^Sc^ is a multimer, while PrP^C^ is monomeric [8], [9]. These conformational differences are the only demonstrated structural differences between PrP^Sc^ and PrP^C^ [10]. Detailed mass spectrometric analysis showed they have identical amino acid sequences [11]. No post-translational differences have been found between PrP^Sc^ and PrP^C^: both share one disulfide bond, two or less sugar antennae and a single glycophosphatidylinositol (GPI) anchor [12]. The composition of the sugar antennae and the GPI anchor vary similarly in both PrP^Sc^ and PrP^C^ [13]. On the other hand, the conformational change and consequent aggregation makes PrP^Sc^ insoluble in non-denaturing detergents and partially resistant to PK digestion [1]. Thus, treatment of a sample with 50 µg/ml of PK for 1 hour at 37°C completely destroys PrP^C^, while, typically, PrP^Sc^ is partially cleaved at the amino terminal portion, leaving a PK-resistant core termed PrP 27–30.

In 1998 Safar *et al.* reported the existence of a subset of PrP^Sc^ molecules that are completely degraded by PK, which hence were termed, PK-sensitive PrP^Sc^ (sPrP^Sc^) [14]. Tzaban *et al.* later demonstrated for the first time that prion-infected tissues contain sPrP^Sc^ molecules that form low molecular weight aggregates [15]. These authors subjected brain homogenates from scrapie-infected animals to sucrose gradients, and found that PrP^Sc^ was distributed in a continuum of aggregation sizes. The more dense fractions, corresponding to larger multimers, were PK-resistant, whereas the intermediate fractions, corresponding to smaller multimers, were not. It has also been described that as much as 90% of total PrP^Sc^ in the brains of individuals who had died as a consequence of Creutzfeldt-Jakob disease (CJD) was estimated to be sPrP^Sc^ [16].

Different studies on other protein misfolding diseases, such as Alzheimer's disease, suggest that large amyloid fibrils may be a means of protecting the host by sequestering the smaller and more toxic multimers as larger less toxic fibrils [17]. In the case of prion diseases, infectivity studies of the different sized fractions of hamster PrP^Sc^ revealed a several-fold increase in infectivity for non-fibrillar particles with masses equivalent to 14–28 PrP molecules [18]. These PrP^Sc^ particle sizes correspond to the sPrP^Sc^ fraction according to Tzaban and our own group's characterization using sucrose gradient ultracentrifugation [15], [19]. Although the definition of sPrP^Sc^ is operational, a question arises: are sPrP^Sc^ and rPrP^Sc^ two populations with different conformations or simply different sized multimers with the same conformation?

To address this question, we investigated first the infectivity of the sPrP^Sc^ *vs.* the PK-resistant (rPrP^Sc^) fraction of hamster PrP^Sc^ (263K strain). sPrP^Sc^ was further characterized by limited proteolysis and mass spectrometry. Then, PrP^Sc^ infectivity and strain characteristics were assessed by inoculation into Syrian hamsters and comparison of the resulting incubation period and lesion distribution with that obtained after inoculation with either total PrP^Sc^ or the PK-resistant fraction of PrP^Sc^. The obtained results are reported here.

## Results

**Figure 1:**
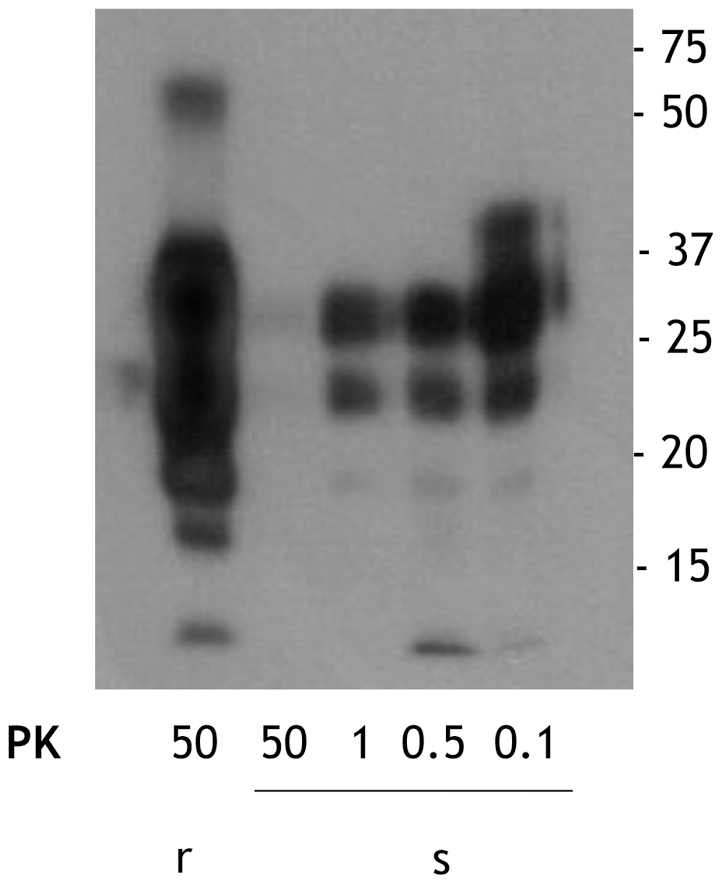
Comparison of the PK resistance of rPrP^Sc^ and sPrP^Sc^ with varying amounts of PK. The PrP^Sc^ fractions, rPrP^Sc^ and sPrP^Sc^, were isolated (see Materials and Methods) and treated with the indicated concentrations of PK and analyzed by WB (probed with mAb 3F4).

We obtained sPrP^Sc^ and rPrP^Sc^ (263K ) fractions from our starting total PrP^Sc^ by our previously described ultracentrifugation-based method [19]. When these fractions were subjected to PK digestion for 1 h at 37°C, virtually complete degradation of sPrP^Sc^ occurred after treatment with 50 µg/ml of PK whereas partial resistance occurred with lower concentrations of the enzyme (Figure 1). In contrast, rPrP^Sc^ was much more resistant to PK under these conditions (Figure 1). These results fully agree with our previously published work [19]. The sPrP^Sc^, rPrP^Sc^, purified PrP^Sc^, and the unpurified PrP^Sc^ material was used in our subsequent experiments.

**Figure 2:**
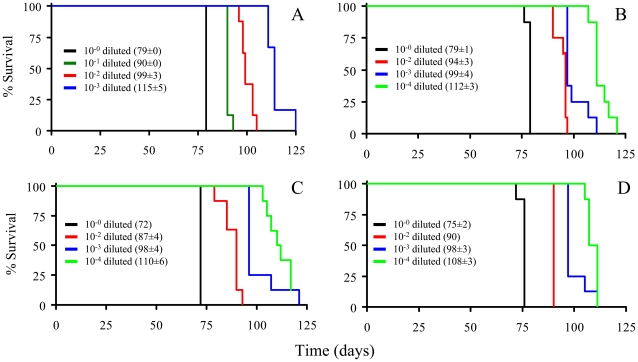
Kaplan-Meier estimate graphs for dilutions of four prion preparations (263K hamster-adapted scrapie). The unpurified PrP^Sc^-containing 0.1% brain homogenate (A); the purified prions from a brain homogenate (B); the sPrP^Sc^ fraction (C); and the rPrP^Sc^ fraction (D). All four undiluted samples contained approximately similar amounts of PrP. Only seven of the eight animals got sick after inoculation with the 10^−4^ dilution of sPrP^Sc^ fraction; the eighth remained healthy.

We performed a bioassay of normalized preparations of four prion isolates, purified PrP^Sc^, sPrP^Sc^, rPrP^Sc^ and the unpurified PrP^Sc^-containing 0.1% brain homogenate. The amount of PrP present in each sample was determined by a mass spectrometry-based quantitative method [20]. The amount of residual PrP^C^ present in the purified samples represents a negligible contribution [21]. Each of the preparations was then diluted, so that each contained approximately similar amounts of PrP. In addition, three dilutions of each of the four normalized samples were prepared: 1/10, 1/100, and 1/1,000 for the 0.1% brain homogenate sample and 1/100, 1/1,000, and 1/10,000 for the other three ones. Each of these dilution series (16 in total) was inoculated into a set of eight Syrian hamsters (LVG) (128 total). The dates of the appearance of clinical signs and the date of euthanization at terminal disease were recorded [22]. These data were plotted as a Kaplan-Meier estimate graph (Figure 2) for each dilution of each preparation. For further clarity, they are also presented in table form (Table S1).

Animals inoculated with the undiluted sPrP^Sc^ preparation got sick sooner than those inoculated with any of the other three undiluted preparations (P<0.01). By the 1/1000 dilution all of the hamsters inoculated with the purified PrP^Sc^ (sPrP^Sc^, rPrP^Sc^, and total purified PrP^Sc^) had a similar incubation time, which indicates a roughly similar infectivity. When the rPrP^Sc^ and purified PrP^Sc^ preparations were diluted 1/10,000 all of the animals became sick. In contrast, of the eight animals inoculated with the 1/10,000 dilution of sPrP^Sc^, only seven became infected. Even after 240 days, the eighth animal showed no clinical signs and there was no detectable PrP^Sc^ in its brain by mass spectrometry-based analysis [20]. Paradoxically the sPrP^Sc^ shows a shorter incubation time in the undiluted preparation (Table S2). Upon dilution of the sPrP^Sc^ preparation the incubation time disproportionately increases compared to the other purified preparations (purified PrP^Sc^ and rPrP^Sc^). At this point, it was not clear if these differences were due to the possibility that our isolation procedure facilitates the isolation of different prion strains, as has been described by Bessen and Marsh [23], or if these differences were caused by kinetic factors, due to the smaller size of the PrP^Sc^ multimers present in the sPrP^Sc^ fraction [15], [19].

These results show that sPrP^Sc^ is infectious, and that its infectivity is comparable to that of rPrP^Sc^. We wanted to see how sPrP^Sc^ behaved after PK treatment. We took a sample of sPrP^Sc^ and divided it into two portions. One was untreated and the other was digested with PK (50 µg/ml of PK; 37°C; 1 h). These samples were inoculated (*ic*) into two sets of six hamsters. The disease course in both sets was observed. In the group inoculated with PK-treated sPrP^Sc^, animals succumbed 15 days later than in the untreated group (Figure S1). This time interval corresponds to a 100 fold dilution of the sPrP^Sc^ fraction (Figure 2). Granting that only an approximate correlation between incubation times and titers can be surmised in our experimental conditions, given that we did not perform a full calibration [20], this result suggests that PK destroyed approximately 99% of the infectivity present in the sPrP^Sc^ fraction. Unlike the unpurified 0.1% brain homogenate, where the loss of infectivity upon treatment with PK is much greater (∼99%) than the loss of WB signal (∼70%) [24], the loss of signal and loss of infectivity are proportionately large (∼99%) with the sPrP^Sc^ fraction (Figure S1).

To assess whether sPrP^Sc^ is also infectious via the *ip* route, we inoculated intraperitoneally two groups of animals with equal amounts of sPrP^Sc^ and rPrP^Sc^, respectively. Both groups succumbed to infection, and although not statistically significant, animals inoculated with the sPrP^Sc^ fraction showed an incubation time slightly shorter (116±9 dpi) than the ones inoculated with the rPrP^Sc^ fraction (123±14 dpi) (Figure S2). As expected, animals inoculated *ip* survived longer than those inoculated *ic* (intracerebral) [25], [26].

After determining that there were phenotypic differences (incubation times) between sPrP^Sc^ and a complete mixture of PrP^Sc^, we explored the possibility that there were structural differences between these two PrP^Sc^ fractions. We have previously reported that, in addition to the well-known N-terminal PK cleavage sites, around position G-90, there are other additional cleavage sites [27]. In that study, we prepared a cleavage map for total PrP^Sc^ after treatment with PK and then treatment with NTCB. The N-terminal cleavage sites include: residues 86 (G-86→D-178), 90 (G-90→D-178), 92 (G-92→D-178), 98 (Q-98→D-178), and 101 (K-101→D-178). Additional cleavage sites comprise residues 117 (A-117→D-178), 119 (G-119→D-178), 135 (S-135→D-178), 139 (M-139→D-178), 142 (G-142→D-178), and 154 (M-154→D-178) ([27] and Figure S3). We used an analogous approach to prepare a cleavage map of sPrP^Sc^. Briefly, sPrP^Sc^ was digested with 1 µg/ml of PK for 30 minutes and subsequently treated with NTCB. This reagent cyanylates free cysteines and NaOH is subsequently used to cleave amide bonds at the N-terminal side of the modified cysteine residues [27], [28]. Given the difficulty in identifying the GPI anchor or sugar-containing peptides by mass spectrometry, only those free of sugar moieties were analyzed. The NTCB reagent cleaves at the cysteine residue at position 179, thereby separating the GPI-anchor and glycosylated portion of the protein from the set of amino-terminal truncated peptides. These peptides contain no post-translational modifications and were analyzed by MALDI-TOF.

**Figure 3:**
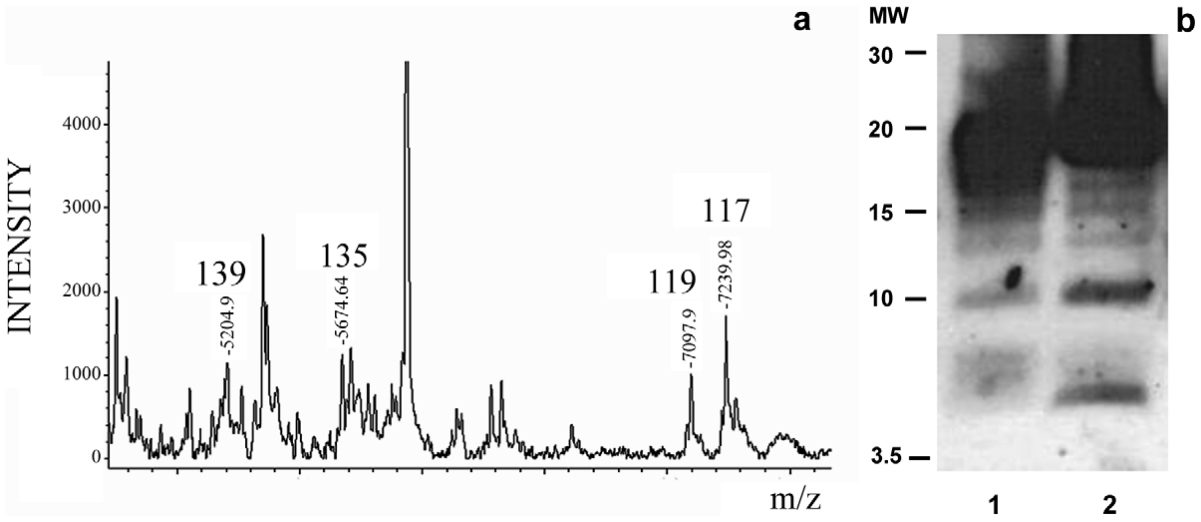
Limited proteolysis of sPrP^Sc^. a: MALDI-TOF spectrum of sPrP^Sc^ treated with 1 µg/ml of PK. The resulting peptides were denatured with 6 M guanidinium chloride and cleaved with NTCB; **b:** Western blot analysis of total PrP^Sc^ (lane 1) and sPrP^Sc^ (lane 2) treated with 50 and 5 µg/ml of PK, respectively; C-terminal R1 antibody was used to probe the blot.

The MALDI-TOF analysis of the NTCB treated sPrP^Sc^ fraction showed cleavage sites identical to the ones previously described for total PrP^Sc^, namely, 117, 119, 135, and 139 (Figure 3a). Curiously, the more abundant peptides from the classic N-terminal PK-cleavage sites (86, 90, and 92) are present in the total PrP^Sc^ samples [27], but not in the sPrP^Sc^ fraction (Figure 3). This suggests that the N-terminal portion of the PrP molecules present in the sPrP^Sc^ fraction might be more exposed and therefore more susceptible to PK digestion than is the N-terminal portion present in the complete mixture of PrP^Sc^. However, it should be remembered that MALDI-TOF is not a quantitative technique and that the signal response of larger peptides, such as (G-86→D-178), or (G-90→D-178), is poorer than that of smaller ones. Indeed, cleavages at around position 90, and perhaps slightly more amino-terminal sites, are prominent in sPrP^Sc^ as shown by WB analysis (*vide infra*).

The similarity of the PK-cleavage pattern between sensitive and total PrP^Sc^ is also evident when comparing the digested samples by Western blot assay using a C-terminal antibody after PK-treatment and deglycosylation (Figure 3b). Besides the intense band corresponding to cleavages around position 90, 6–7 additional lower molecular weight bands are seen in both cases, as we have previously described for total PrP^Sc^ [27]. Although we do not know the identity of each band, the apparent molecular weight of some of them could correspond with cleavages seen in MALDI-TOF. While the relative intensities of some of the bands vary between sPrP^Sc^ and total PrP^Sc^, (the main band centered at 19–20 kDa smears a bit more into the 20–21 kDa region in the sPrP^Sc^ sample, the ∼17 kDa band is more intense in total PrP^Sc^, bands around 15, and finally, bands at ∼10 and ∼6 kDa are more intense in sPrP^Sc^), the overall pattern is very similar.

**Figure 4:**
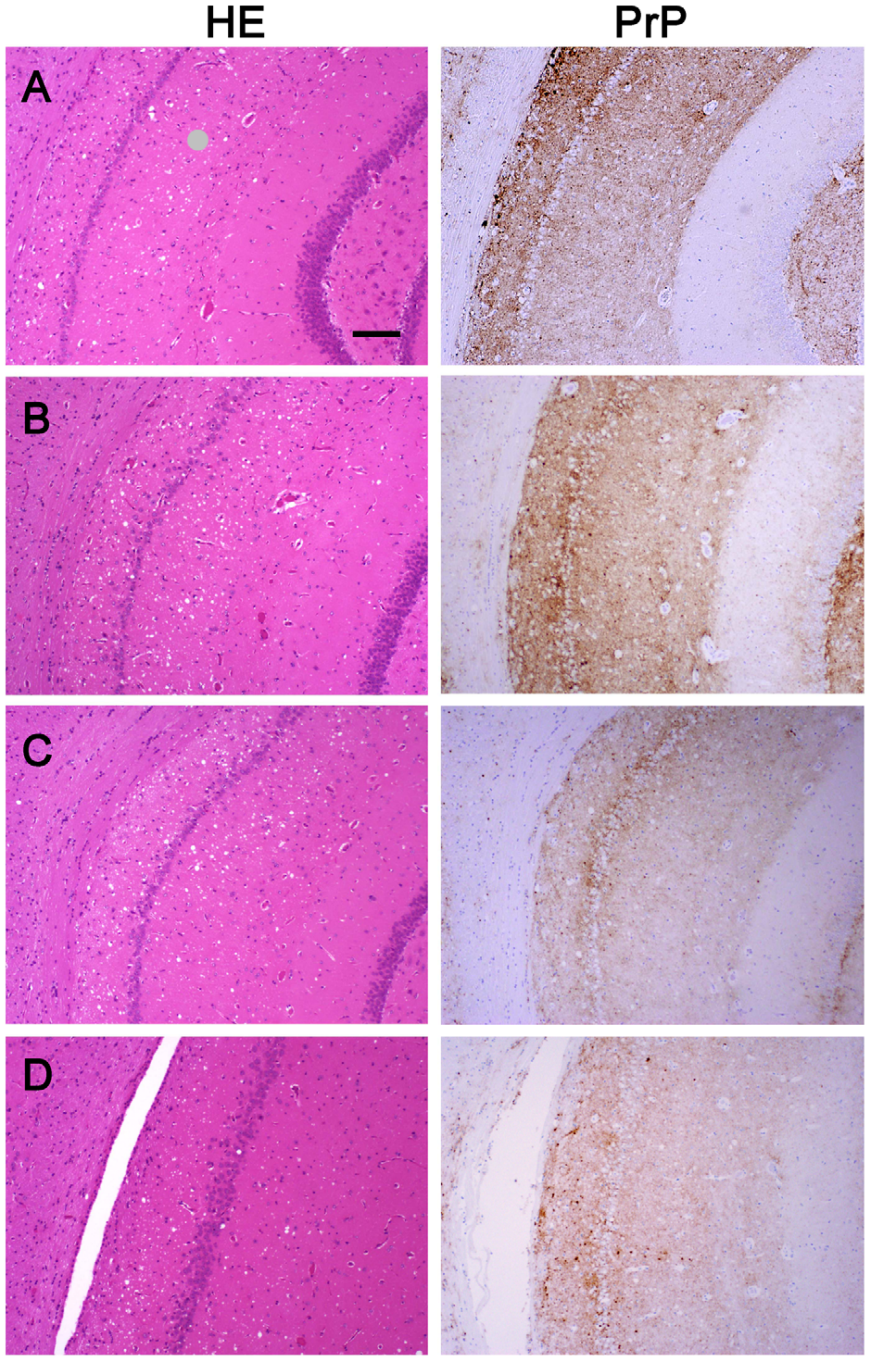
Histopathological and immunohistochemical analyses. Sections from the brain at the level of the hippocampus of hamsters inoculated (*ic*) with either the sPrP^Sc^ fraction (A); the rPrP^Sc^ fraction (B); purified prions from a brain homogenate (C); or the unpurified 10% brain homogenate (D). Each section was stained with hematoxylin and eosin (HE) or prepared for immunohistochemical analysis using the 3F4 antibody (PrP).

**Figure 5:**
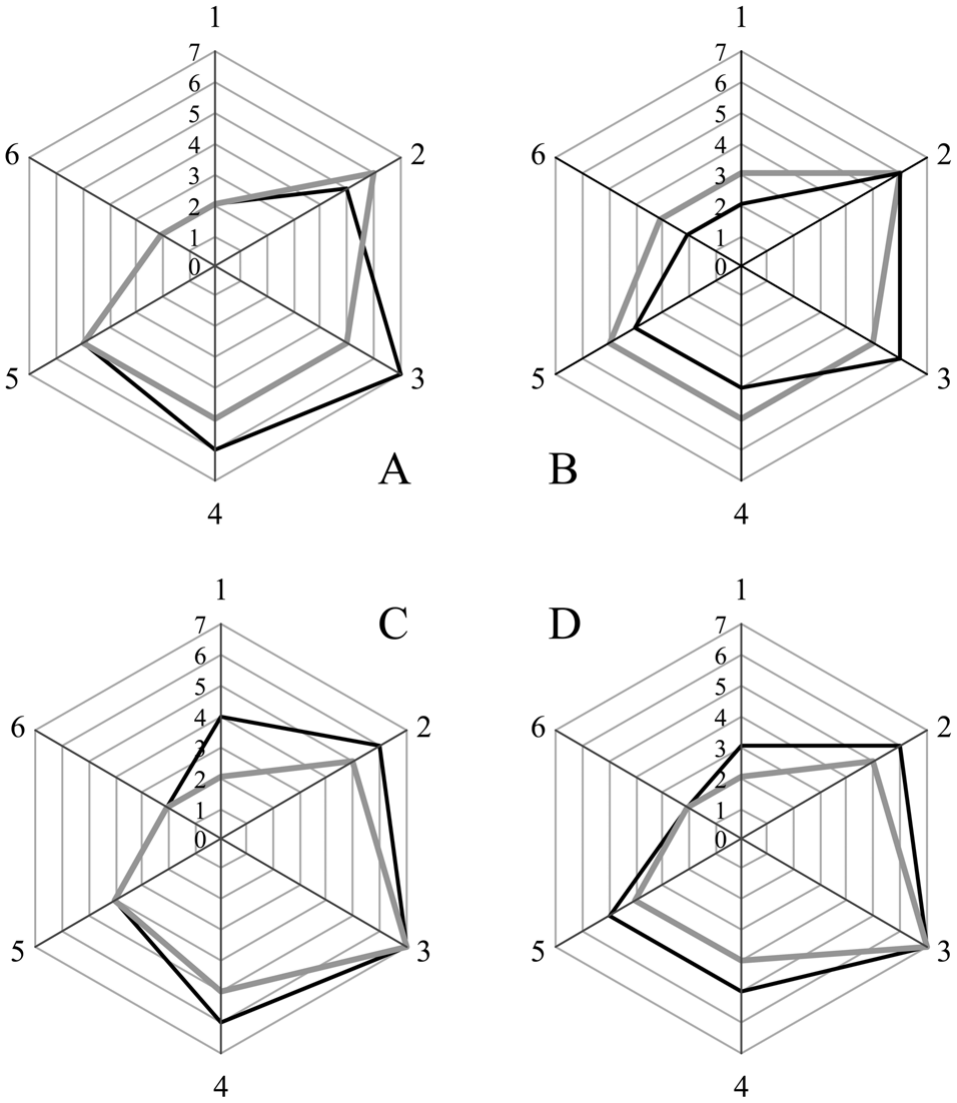
Radar plot of the lesion profiles (scored 0–7) from six regions (1–6) of the brain from hamsters inoculated with prions. Lesion profile scores (*vide infra*) reflect the spongiform degeneration and PrP^Sc^ deposition. Samples from the brains of two hamsters (solid grey or black lines) inoculated with the unpurified PrP^Sc^-containing 0.1% brain homogenate (A), the purified prions from a brain homogenate (B), the sPrP^Sc^ fraction (C), or rPrP^Sc^ fraction (D). Six regions were examined: cerebellum (1), medial thalamus (2), hippocampus (3), cortex over thalamus (4), cortex-medial (5), and cerebral peduncles (6).

We wanted to ensure that in fractionating PrP^Sc^, we had not inadvertently isolated a new prion strain. We performed comparative histopathological and immunohistochemical studies on the left hemisphere of brains from hamsters infected with each of the four PrP^Sc^ preparations. Spongiform change and PrP deposition were detected in all cases with no detectable differences in the PrP^Sc^ distribution or the morphology of the PrP^Sc^ aggregates among all four groups (Figure 4). The PrP^Sc^ appeared as diffuse as well as small, punctate aggregates. To assess whether there were differences in the brain regions targeted, lesion profiles of the hamsters inoculated with four different prion preparations was also performed. We compared the spongiform degeneration and PrP^Sc^ deposition in 6 brain regions. The results of this lesion profile analysis suggest that the isolation procedure did not yield different strains (Figure 5).

Western blot analysis of brain homogenates from the four inoculated groups showed the presence of PK resistant PrP in all groups. No differences in band patterns were detected (Figure S4). The relative amount of sPrP^Sc^ in each group was assessed by subtracting the amount PrP^Sc^ present in an aliquot digested with PK from the amount of PrP^Sc^ present in an untreated aliquot. The amount of PrP^Sc^ was determined by a previously reported mass spectrometry-based method [20]. The proportion of sPrP^Sc^ present in the brains of animals inoculated with either unpurified brain homogenate, purified prions, rPrP^Sc^ or sPrP^Sc^ was 0.3, 0.3, 0.6 or 0.5, respectively (means of duplicate analyses). In our previous work, we determined that sPrP^Sc^ accounted for between 35 and 60% of the total PrP^Sc^ present in the sample [19]. Thus the relative amount of sPrP^Sc^ in all groups is similar to that present in an unpurified brain homogenate.

We chose to use the drowsy (Dy) strain of hamster-adapted prion disease in order to compare the results with those obtained from 263K-derived prions. The phenotype of the Dy strain is very different from that of 263K [29]. It has a much longer incubation period and the typical symptoms are progressive lethargy and kyphosis [23]. The Dy strain is highly susceptible to PK digestion [30]. We wondered whether such susceptibility correlated with a higher ratio of sensitive to resistant fraction and if this would provide some insight into the nature of sPrP^Sc^ isolated from the 263K strain.

We isolated Dy sPrP^Sc^ using our method of isolating PK-sensitive prions (Figure S5) [19]. The sensitive fraction of Dy was shown to constitute approximately half of the total PrP^Sc^, whereas in 263K, the analogous PK-sensitive fraction represents a considerably lower proportion of the total PrP^Sc^ (Figure S6). This could partially explain why Dy is more PK-susceptible than 263K. These results agree with those of Safar *et al.* who reported that different strains of hamster scrapie prions contain different ratios of sensitive to resistant PrP^Sc^ [14]. It should be noted that, although our PrP^Sc^ purification protocol involves sonication and therefore fragmentation of large aggregates, the sonication conditions used in 263K and Dy PrP^Sc^ isolation were the same.

**Figure 6:**
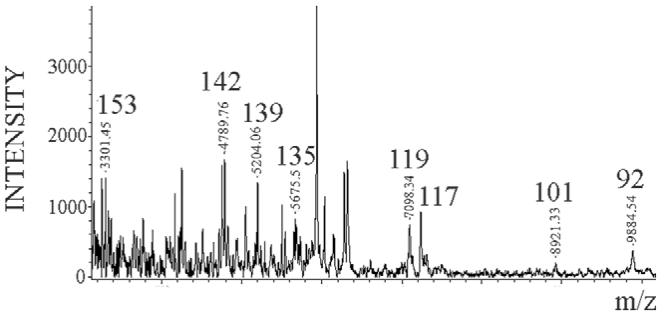
Limited proteolysis of Dy sPrP^Sc^. Dy sPrP^Sc^ was treated with 0.1 µg/ml of PK. The resulting peptides were denatured with 6 M guanidinium chloride and cleaved carboxy-terminally at position C_179_ with NTCB, and analyzed by MALDI-TOF.

The MALDI-TOF spectrum of the Dy sensitive fraction after digestion with 0.1 µg/ml of PK and NTCB cleavage showed the same internal cleavage sites as in 263K (117, 119, 135, and 139) plus others (101 (K-101→D-178) and 92 (G-92→D178)) (Figure 6) also previously described for total PrP^Sc^ (Dy strain) (Figure S3 and [27]).

## Discussion

Most prions have a degree of resistance to proteinase K digestion. Digestion of prions with proteinase K yields a characteristic prion core referred to as PrP 27–30 and a loss of infectivity that is disproportionate to the loss of protein. As researchers examined hamster strains they realized that not all strains of prions were equally resistant to proteinase K digestion, as is the case with the hyper (Hy) and drowsy (Dy) strains of hamster-adapted transmissible mink encephalopathy [23], [30]. Other researchers observed a similar phenomenon with other prions [31], [32], [33], [34]. Later Safar showed that PrP^Sc^ contained both a proteinase K sensitive and proteinase K resistant fraction [14]. These results indicated that PrP^Sc^ can be both infectious and proteinase K sensitive and that these phenotypes are a general characteristic of prions.

Since Safar *et al.* reported the existence of a PK-sensitive fraction in PrP^Sc^, little has been published about its structural characteristics and infectious properties [14]. Although its infectivity has been suggested [14], [35], it had not been proven. In this work we demonstrate that the sensitive fraction of PrP^Sc^ is roughly as infectious as the resistant one. Highly significant is the fact that both fractions presented similar incubation times even when the respective inocula were diluted 1000 times. It has been shown that when PrP^Sc^ was fragmented by sonication, infectious titer was reduced because the rate of PrP^Sc^ clearance from the brain increased [36]. PrP^Sc^ is rapidly cleared from the brain once inoculated and the rate of clearance is influenced by the particle size [37]. Our results indicate that both rPrP^Sc^ and sPrP^Sc^ fractions have the same degree of infectivity indicating that the aggregation state of PrP^Sc^ does not affect its infectivity. This is in contrast with a previous report where the majority of sPrP^Sc^ was found to be not infectious when measured by the scrapie cell assay [38]. Also, Weber *et al.* found that extended sonication of PrP^Sc^ was associated with a loss of infectivity as measured by the prolongation of the incubation time [39]. In this case some PrP^Sc^ may be degraded during sonication. In our case, total PrP^Sc^ is isolated first, when sonication occurred, and then both fractions are separated at an intermediate centrifugation speed with no further sonication. On the other hand, hamster 263K ( = Sc237) prions did not show altered incubation times when the prion rods were fragmented by sonication into spherical particles [40].

Several explanations may account for the similarity in infectivity values in sPrP^Sc^ and rPrP^Sc^. Namely, common and highly infectious species are present in both fractions and thus are subject to the same rate of clearance. Another possibility is the presence of one or more cofactors that copurify with both fractions of PrP^Sc^. PrP 27–30 rods that dissociated into much smaller detergent-lipid-protein complexes and liposomes led to a 100-fold increase in infectivity as measured by endpoint titration [41]. Perhaps the binding of PrP^Sc^ to the cell membrane increases the efficiency of disease transmission from cell to cell.

Limited proteolysis studies on the sPrP^Sc^ fraction of 263K and Dy indicate that, as in total PrP^Sc^, the sensitive fraction is also composed of alternating PK-resistant stretches interspersed with PK-susceptible stretches. The relatively small variations in intensity of some of the PK-resistant bands detected by SDS-PAGE (Fig. 3b), could be interpreted as corresponding to differences in accessibility of the PK-resistant stretches involved, but the global emerging picture is one of a shared basic structural organization.

This result emphasizes the fact that the difference between sensitive and resistant PrP^Sc^ seems to be the previously described different size of the aggregates and not the conformation of their respective aggregates [15], [19]. Our data provide information on the N-terminal half of the protein only, therefore we cannot rule out differences in the C-terminal portion.

As we mentioned in the introduction, the sensitive fraction of 263K varies between 20–40% of the total PrP^Sc^. Despite this variability, the amount of sensitive PrP^Sc^ obtained for 263K was always less than that for Dy. This result, previously reported by Safar *et al.* [14], would indicate that a specific ratio of sensitive to resistant exists for each strain. This author also reported that the incubation time of a given prion strain is directly proportional to the level of protease-sensitive PrP^Sc^ [14]. According to Deleault *et al.*, variations in the sPrP^Sc^∶rPrP^Sc^ ratio between different prion strains appear to be an incidental product rather than a strain property [42]. Nevertheless, we want to point out that for hamster-adapted prion diseases the PK-susceptibility seems to directly correlate with the proportion of sPrP^Sc^ present in the strain.

In summary, we have shown that sPrP^Sc^ is fully infectious, and that its infectivity can be reduced by approximately 99% by treatment with PK (50 µg/mL; 1 hr). sPrP^Sc^ appears to share the same basic architecture with rPrP^Sc^, as judged by the generation of similar fragmentation patterns by limited proteolysis with PK as seen in the WB and MALDI analysis. This, together with the fact that sPrP^Sc^ induces a disease with signs and histopathological and lesion profiles that are essentially identical to those induced by total or rPrP^Sc^, strongly suggests that sPrP^Sc^ differs from rPrP^Sc^, as previously shown, just in its smaller size, *i.e.*, being made up of fewer PrP units [15], [19]. On the other hand, the fact that different prion strains are characterized by different relative ratios of sPrP^Sc^∶rPrP^Sc^ suggests that sPrP^Sc^ might play an important role in key properties of strains. Finally, sPrP^Sc^, which is soluble in detergent-containing solutions, might prove to be useful in studies aimed at elucidating the structure of PrP^Sc^.

## Materials and Methods

### Ethics statement

Animal experiments were carried out in accordance with the recommendations contained in the Guide for the Care and Use of Laboratory Animals of the National Institutes of Health. The procedures were governed by a protocol that was approved by the Institutional Animal Care and Use Committee of the United States Department of Agriculture, Agricultural Research Service, Albany, CA (Protocol Number: P-10-3). All surgery was performed under isoflurane anaesthesia, and all efforts were made to minimize the suffering of the animals. The small numbers of experiments carried out at the University of Santiago de Compostela were approved by the University Ethics Committee, in accordance with the European Union Council Directive 86/609/EEC.

### Isolation of total PrP^Sc^ and sPrP^Sc^

PrP^Sc^ was isolated from brains of terminally ill Syrian hamsters infected intracranially (*ic*) with the 263K strain of scrapie using a slightly modified version of the procedure of Diringer *et al.* [43]. A cocktail of protease inhibitors (Complete, Roche Diagnostics, Penzberg, Germany) at a final concentration of 1× was used in all buffers throughout the procedure up to the penultimate pellet, as defined in the mentioned study [43]. The final pellet was resuspended in 20 mM Tris (pH 8.5) containing 1% sarkosyl and no protease inhibitors, at concentrations between 1–2 µg/µl. The stock suspension thus prepared was aliquoted and frozen until further use; its purity was assessed by SDS-PAGE with Coomassie blue staining, and estimated to be approximately 80%. sPrP^Sc^ was isolated from total PrP^Sc^ by ultracentrifugation at an intermediate speed [19]. A 50–150 µl portion of the PrP^Sc^ stock was homogenized by application of 3 sonication pulses of 1 s each, and spun in a Beckman TLX ultracentrifuge (Beckman, Fullerton, CA) using a TLA-120-1 rotor at 40,000 rpm (56,806 g) for 2 hours at 20°C. The supernatant was collected and the pellet was resuspended by sonication in a volume of 20 mM Tris (pH 8.5) containing 1% sarkosyl equivalent to that of the supernatant. Fractions of supernatant and pellet were treated with 50 µg/ml of PK at 37°C during 1 hour; the reaction was terminated with 2 mM Pefabloc (Fluka, St. Louis, MO), and a fraction subjected to SDS-PAGE and either stained with Coomassie brilliant blue or transferred to a PVDF membrane (Immobilon-P, Millipore, Billerica, MA, USA) and analyzed by Western blotting using mAb 3F4 (Dako, Glostrup, Denmark). The supernatant fraction is sPrP^Sc^, and the pellet fraction, PK-resistant PrP^Sc^ (rPrP^Sc^). The concentration of PrP^Sc^ was estimated in each of these samples and in brain homogenates. PrP^Sc^ was isolated by ultracentrifugation using the method of Bolton et al. [44], with slight modifications [20]. The resulting pellets were dissolved in 200 µL of 6 M guanidinium chloride and allowed to stand at RT for 24 hours to inactivate the PrP^Sc^ [45]. The inactivated prion solutions were precipitated with methanol and subjected to analysis by mass spectrometry using previously described methods [20], [21]. Briefly, pellets were dissolved in 0.01% aqueous beta-octylglucopyranoside (BOG) and [^13^C_5_,^15^N]-VVEQMCTTQYQK added as internal standard. The samples were then sequentially reduced, alkylated and digested with trypsin. NanoLC/MS/MS was carried out with an Applied Biosystems (ABI/MDS Sciex, Toronto, Canada) model 4000 Q-Trap instrument equipped with a nano-electrospray ionization source. The mass spectrometer was operated in multiple reaction monitoring (MRM) mode, alternating between detection of VVEQMCTTQYQK (precursor ion m/z of 757.8, product ion of m/z 171.1) and [^13^C_5_,^15^N]-VVEQMCTTQYQK (precursor ion m/z of 760.8, product ion of m/z 177.1). Quantitation was done with the Intelliquan quantitation algorithm of Analyst 1.4.1 software (Applied Biosystems) using default parameters [20], [21].

### Bioassays

Four prion isolates, 0.1% brain homogenate, purified PrP^Sc^, sPrP^Sc^, and rPrP^Sc^, were diluted to yield four corresponding solutions containing 1.5±0.4 ng of PrP per 50 µL of sample, except in the case of the 0.1% brain homogenate, which contained 0.7 ng of PrP per 50 µL of sample (vide supra). A portion of each of these normalized solutions was diluted 100-fold, 1,000-fold and 10,000-fold into sterile phosphate buffered saline (PBS) to yield a set of four dilutions (including the undiluted material) for each of the prion isolates; in the case of the 0.1% brain homogenate sample, dilutions were 10-fold, 100-fold, and 1,000-fold. Fifty microliters of each dilution were ic inoculated into the right cerebral hemisphere of a four week old anesthetized female hamster. Eight animals were used per isolate/dilution.

The 128 animals were placed in cages (2 per cage) and observed for clinical signs. The first clinical sign to be observed and its date of occurrence recorded was an exaggerated startle response. A progression of clinical signs followed, including an exaggerated startle response that became more pronounced over time, ataxic gait whose severity increased with time, limb rigidity, characteristic head bobbing, and progressive lethargy. When the animals could no longer be roused from their recumbency to feed and drink water, they were humanely euthanized. The incubation time was measured (in days) as the time between inoculation and euthanization.

After 240 days one of the inoculated animals (sPrP^Sc^; 10,000-fold dilution) animals remained healthy. The animal was euthanized and its brain removed. The brain was analyzed for the presence of PrP 27–30 by mass spectrometry [20]. There was no evidence of PrP 27–30 present in the brain.

In a separate experiment, two groups of 6 animals were inoculated *ic* with either 50 µl of a sample containing ∼40 ng of sPrP^Sc^ or the same volume of sPrP^Sc^ treated with 50 µg/ml of PK for 1 h at 37°C, followed by quenching of PK with 2 mM Pefabloc. Infection progression was monitored as described above, and approximate infectivity titers of the PK-treated and untreated sPrP^Sc^ samples were estimated by incubation period assay according to the procedure of Prusiner *et al.* [22].

Finally, two groups of 6 animals were intraperitoneally (*ip*) inoculated with 150 µl volumes of sPrP^Sc^ and rPrP^Sc^ prion isolates containing ∼125 ng of PrP each, and the course of infection observed as described above. The amount of PrP^Sc^ in these two samples was calculated approximately by comparison with a recombinant Syrian hamster (rSha) PrP (90–231) standard (a generous gift of Giuseppe Legname) after deglycosylation with PNGase F (New England Biolabs, Ipswich, MA, USA) and Western blotting with mAb 3F4.

### Histopathology and immunohistochemical stains

Four µm thick sections were cut onto positively charged silanized glass slides and stained with hematoxylin and eosin, or immunostained using an antibody for PrP (3F4). Immunohistochemical stains for PrP were performed entirely on an automated Discovery XT staining apparatus (Ventana Medical Systems, Oro Valley, AZ) using a DAB Map XT Detection Kit (Ventana Medical Systems). Sections were deparaffinised and then washed in distilled water for 5 min. Epitope exposure was performed by heating sections to 100°C in a citrate buffer for 12 minutes. After treatment with protease 2 (Ventana Medical Systems) for 24 minutes, sections were incubated with anti-PrP 3F4 for 60 min and PrP was detected using HRP-conjugated antibodies and a DAB substrate.

### Lesion profiling

We selected 6 anatomic brain regions in accordance with previous strain typing protocols [46], [47] from 2 hamsters per group. We scored spongiosis on a scale of 0–4 (not detectable, mild, moderate, severe and *status spongiosus*) and PrP immunological reactivity on a 0–3 scale (not detectable, mild, moderate, severe). A sum of the two scores resulted in the value obtained for the lesion profile for the individual animal. The ‘radar plots’ depict the scores for spongiform changes and PrP deposition. Numbers correspond to the following brain regions: (1) cerebellum, (2) medial thalamus, (3) hippocampus, (4) medial cerebral cortex dorsal to hippocampus, (5) medial cerebral cortex dorsal to septum, (6) white matter at cerebellar peduncles. Investigators blinded to animal identification performed histological analyses.

### Biochemical characterization and quantitation of PrP^Sc^ in brains of inoculated animals

PrP^Sc^ was detected in brain homogenates of inoculated animals by Western blot using mAb 3F4 (primary antibody) and goat anti-mouse Fc (secondary antibody) (Sigma-Aldrich, St. Louis, MO).

To assess the relative amount of sPrP^Sc^ and rPrP^Sc^ in animals inoculated with the four different inocula described, duplicate PrP^Sc^ samples were isolated from the brains of inoculated hamsters using the method of Bolton *et al.* [44] with slight modifications [20]. The resulting pellets were either dissolved in 200 µL of 6 M guanidinium chloride (Sigma-Aldrich, St. Louis, MO) or resuspended in 0.1% Z 3,14-T-8.5 (0.1% 3-(N,N-dimethylmyristyl-ammonium)propane sulfonate; 20 mM Tris pH 8.5). The 6 M guanidinium chloride solutions were allowed to stand for 24 hours at RT to inactivate the PrP^Sc^ [45]. The samples suspended in 0.1% Z 3,14-T-8.5 were sonicated. After sonication, PK was added to make a final concentration of 5 µg/mL. The PK was permitted to react for 1 hour at 37°C. The reaction was quenched by addition of a sufficient amount of phenylmethylsulfonyl fluoride (PMSF) (Sigma-Aldrich, St. Louis, MO) to achieve a final concentration of 1 mM. Enough solid guanidinium chloride was then added to make a 6 M solution, which was left to stand for 24 hours at RT to inactivate the prions. The inactivated prion solutions were precipitated with methanol and the amount of PrP present in the sample was determined by mass spectrometry (*vide supra*) [20], [21].

### NTCB cleavage reaction of PrP^Sc^ samples

All buffers and reagents were prepared fresh for the 2-nitro-5-thiocyanatobenzoic acid (NTCB) reactions. Between 15–20 µg of purified PrP^Sc^ was reduced in 2 mM DTT for 1 h at 37°C under denaturing conditions (6 M guanidinium chloride and 100 mM Tris buffer pH 8). In the same buffer, samples were cyanylated in 10 mM 2-nitro-5-(thiocyanato)-benzoate (NTCB) for 30 minutes at RT. After methanol precipitation, samples were redissolved in 6 M guanidinium chloride, 100 mM Tris buffer pH 8. PrP^Sc^, specifically cyanylated at the reactive thiols, was cleaved by alkaline hydrolysis with 150 mM NaOH for 15 minutes at 37°C. The NTCB reaction was terminated by addition of trifluoroacetic acid (TFA) to a final pH of 2–3. PrP^Sc^ peptides were isolated from the reaction mixture using C-18 ZipTips (Millipore) according to the manufacturer's instructions. Peptides were eluted from the tips in 10 µl of 50% acetonitrile, 0.1% TFA and used directly for MALDI analysis.

### MALDI mass spectrometry

A 2 µL portion of the PrP^Sc^ peptide sample was mixed with an equal volume of a saturated solution of sinapinic acid in acetonitrile and 0.1% aqueous TFA (1∶2). One microliter of the mixture was spotted onto a Bruker sample plate, allowed to air-dry, and analyzed using a Bruker Autoflex MALDI instrument in linear mode. The laser frequency was 25 Hz; pulsed ion extraction was set at a value of 140–150 ns. Repeated laser shots, typically 25–30, were averaged.

### Analysis of PK-resistant fragments of sPrP^Sc^ and total PrP^Sc^ by Western blot

PK-treated samples were deglycosylated with PNGase F (New England Biolabs, Ipswich, MA, USA) at 37°C for 48 h, according to the manufacturer's instructions, followed by precipitation with ice-cold 85% MetOH. Pellets were boiled in 10 µl of reducing tricine sample buffer (BioRad, Hercules, CA, USA). Tricine SDS-PAGE [48] was carried out using 10–20% Tris-Tricine/Peptide Precast gels (BioRad), in a Criterion electrophoresis system (BioRad). The cathode buffer was Tris-Tricine-SDS buffer (Sigma-Aldrich) and the anode buffer, 100 mM Tris-HCl, pH 8.9. Electrophoresis was performed at a constant voltage of 125 volts for 200 minutes, on ice. After electroblotting on PVDF membranes, these were probed with antibody R1 (a generous gift from Hanna Serban, UCSF), which recognizes residues 226–231. Peroxidase-labeled anti-human antibody was used as the secondary antibody.

## Supporting Information

Figure S1 Loss of infectivity of sPrP^Sc^ upon treatment with 50 µg/ml of PK.
(TIF)

Figure S2 Incubation time of sPrP^Sc^ and rPrP^Sc^ inoculated by the intraperitoneal route.
(TIF)

Figure S3 MALDI-TOF spectrum of PrP^Sc^ treated with 1 µg/ml of PK, showing the presence of cleavage sites. Adapted from Sajnani *et al.* (2008) J Mol Biol 382: 88–98, with permission from Elsevier Ltd. The Boulevard, Langford Lane, Kidlington, Oxford OX5 1GB UK.
(TIF)

Figure S4 Western blots of PrP^Sc^ isolated from the brains of hamsters inoculated with unpurified brain homogenate, purified prions from a brain homogenate, purified rPrP^Sc^, or purified sPrP^Sc^. Lanes 1, 2, and 9, 10: duplicate animals inoculated with unpurified brain homogenate. Lanes 3, 4, and 11, 12: duplicate animals inoculated with purified PrP^Sc^ from a brain homogenate. Lanes 5, 6, and 13, 14: duplicate animals inoculated with rPrP^Sc^. Lanes 7, 8, and 15, 16: duplicate animals inoculated with sPrP^Sc^. The equivalent of 5 mg of brain tissue was loaded in each lane. Blots were probed with mAb 3F4 (primary antibody) and goat anti-mouse Fc (secondary antibody).
(TIF)

Figure S5 Comparison of the PK resistance of Dy rPrP^Sc^ and sPrP^Sc^. Dy PrP^Sc^ fractions, rPrP^Sc^ (r) and sPrP^Sc^ (s), were isolated (see Materials and Methods) and treated with the indicated concentrations of PK and analyzed by WB (probed with mAb 3F4).
(TIF)

Figure S6 Relative amounts of sPrP^Sc^ in 263K and Dy PrP^Sc^. Samples were probed with mAb 3F4.
(TIF)

Table S1 Table of Kaplan-Meier endpoint data. Tabular summary of the Kaplan-Meier estimate graphs for dilutions of four prion preparations (263K hamster-adapted scrapie). The unpurified PrP^Sc^-containing 0.1% brain homogenate (Unpurified brain homogenate); the purified prions from a brain homogenate (purified prions); the PK sensitive prion fraction (sPrP^Sc^); and the PK resistant prion fraction (rPrP^Sc^). The data represents the time from inoculation to euthanization ± SD, as measured in days. Eight animals were used per dilution. Some dilutions were not done (n.d.). Only seven of the eight animals got sick after inoculation with the 10^−4^ dilution of sPrP^Sc^ fraction; the eighth remained healthy.
(DOC)

Table S2 Table of P-values for t-tests. The t-test was used to compare the Kaplan-Meier estimate data for the unpurified prions (0.1% BH), purified prions, PK resistant prions (rPrP^Sc^), and the PK-sensitive prions (sPrP^Sc^). The listed p-value is the result of a comparison of the Kaplan-Meier data for a row with that of a column. Each of the four dilutions (10^−0^, 10^−2^, 10^−3^, and 10^−4^) is in a separate table. A statistically significant difference (Sig) means that the P-value (in parentheses) is greater than or equal to 0.99. A statistically insignificant difference (NSig) means the P-value that is less than 0.99.
(TIF)
