# Supplementary figures and images for: PK-sensitive PrPSc Is Infectious and Shares Basic Structural Features with PK-resistant PrPSc
Source: PLoS Pathog. 2012 Mar 1;8(3):e1002547. doi: 10.1371/journal.ppat.1002547 (PMC3291653; doi:10.1371/journal.ppat.1002547)

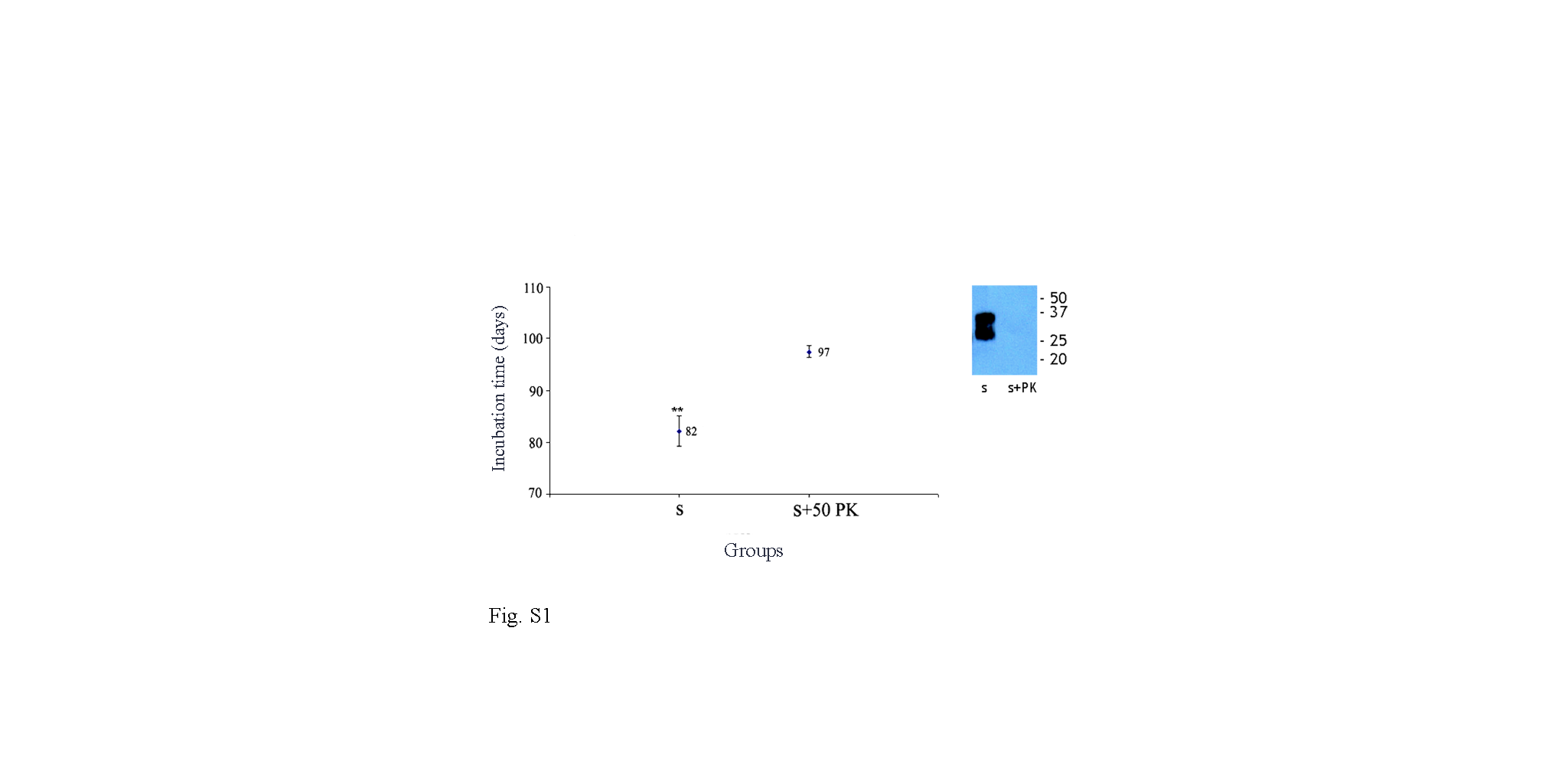

Supplement: Figure S1 — Loss of infectivity of sPrPSc upon treatment with 50 µg/ml of PK. (TIF) [file ppat.1002547.s001.tif]

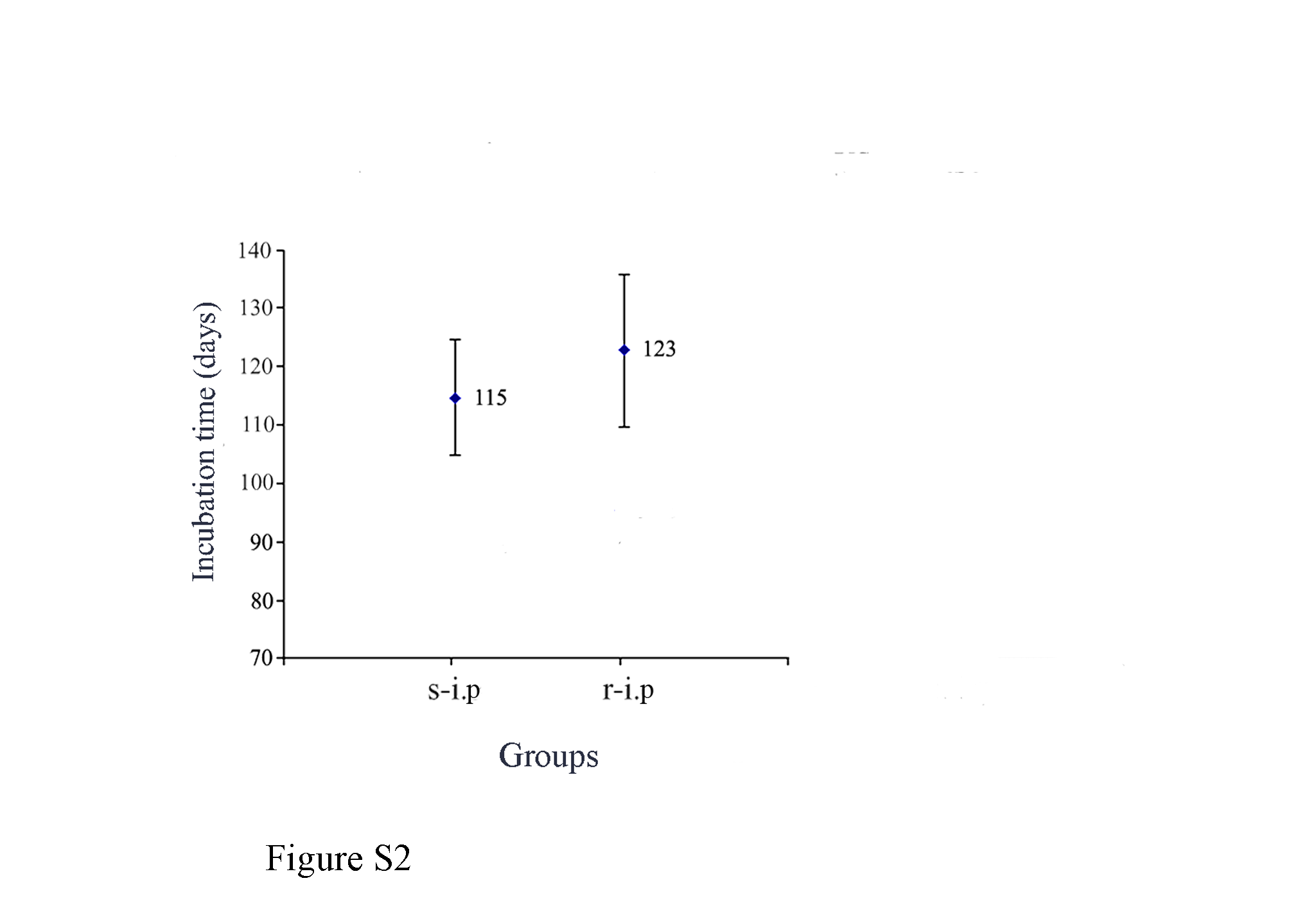

Supplement: Figure S2 — Incubation time of sPrPSc and rPrPSc inoculated by the intraperitoneal route. (TIF) [file ppat.1002547.s002.tif]

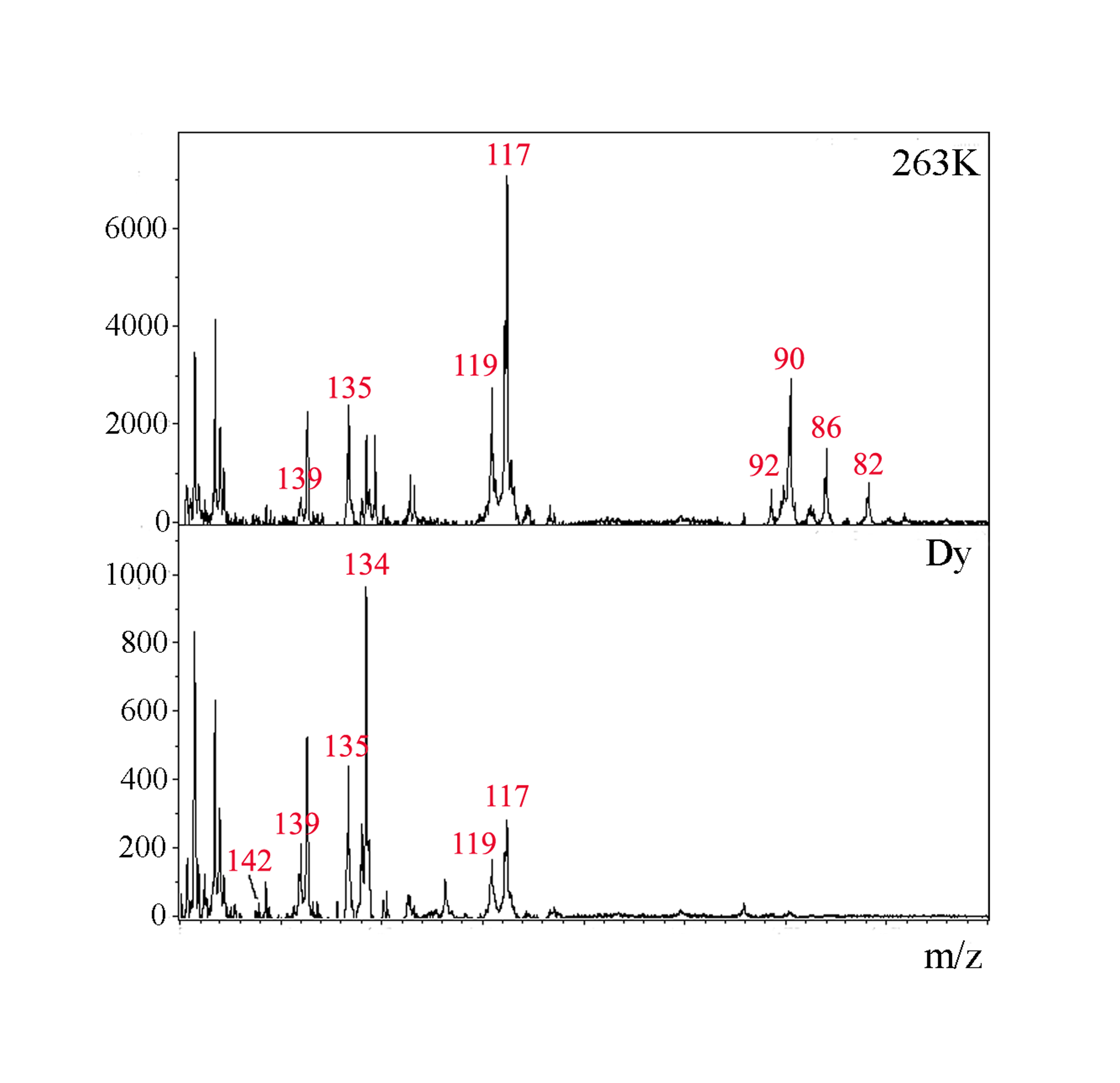

Supplement: Figure S3 — MALDI-TOF spectrum of PrPSc treated with 1 µg/ml of PK, showing the presence of cleavage sites. Adapted from Sajnani et al. (2008) J Mol Biol 382: 88–98, with permission from Elsevier Ltd. The Boulevard, Langford Lane, Kidlington, Oxford OX5 1GB UK. (TIF) [file ppat.1002547.s003.tif]

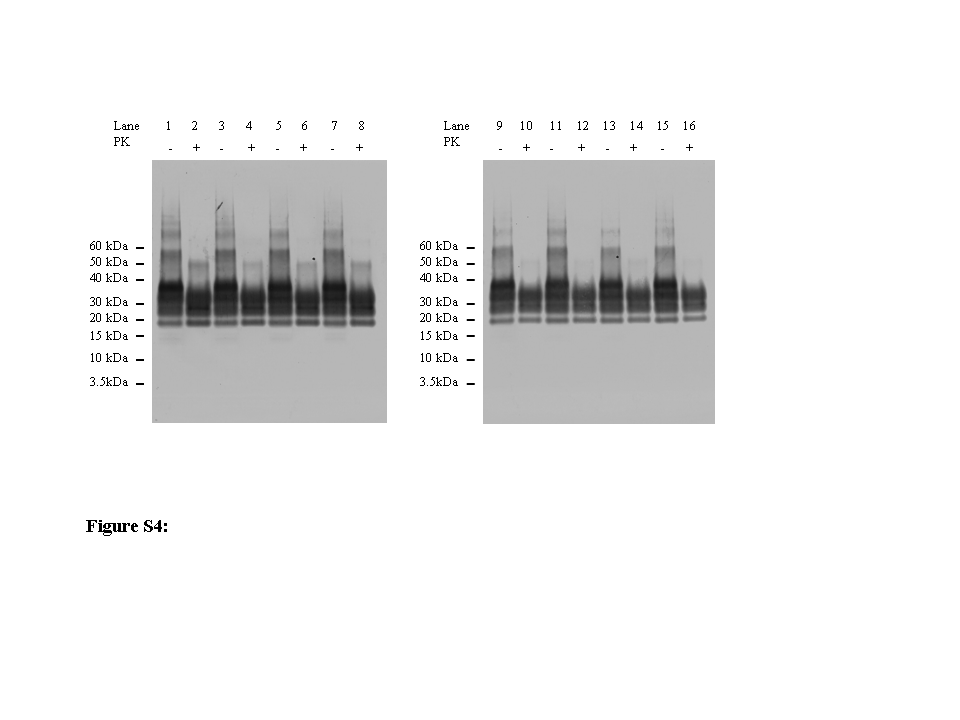

Supplement: Figure S4 — Western blots of PrPSc isolated from the brains of hamsters inoculated with unpurified brain homogenate, purified prions from a brain homogenate, purified rPrPSc, or purified sPrPSc. Lanes 1, 2, and 9, 10: duplicate animals inoculated with unpurified brain homogenate. Lanes 3, 4, and 11, 12: duplicate animals inoculated with purified PrPSc from a brain homogenate. Lanes 5, 6, and 13, 14: duplicate animals inoculated with rPrPSc. Lanes 7, 8, and 15, 16: duplicate animals inoculated with sPrPSc. The equivalent of 5 mg of brain tissue was loaded in each lane. Blots were probed with mAb 3F4 (primary antibody) and goat anti-mouse Fc (secondary antibody). (TIF) [file ppat.1002547.s004.tif]

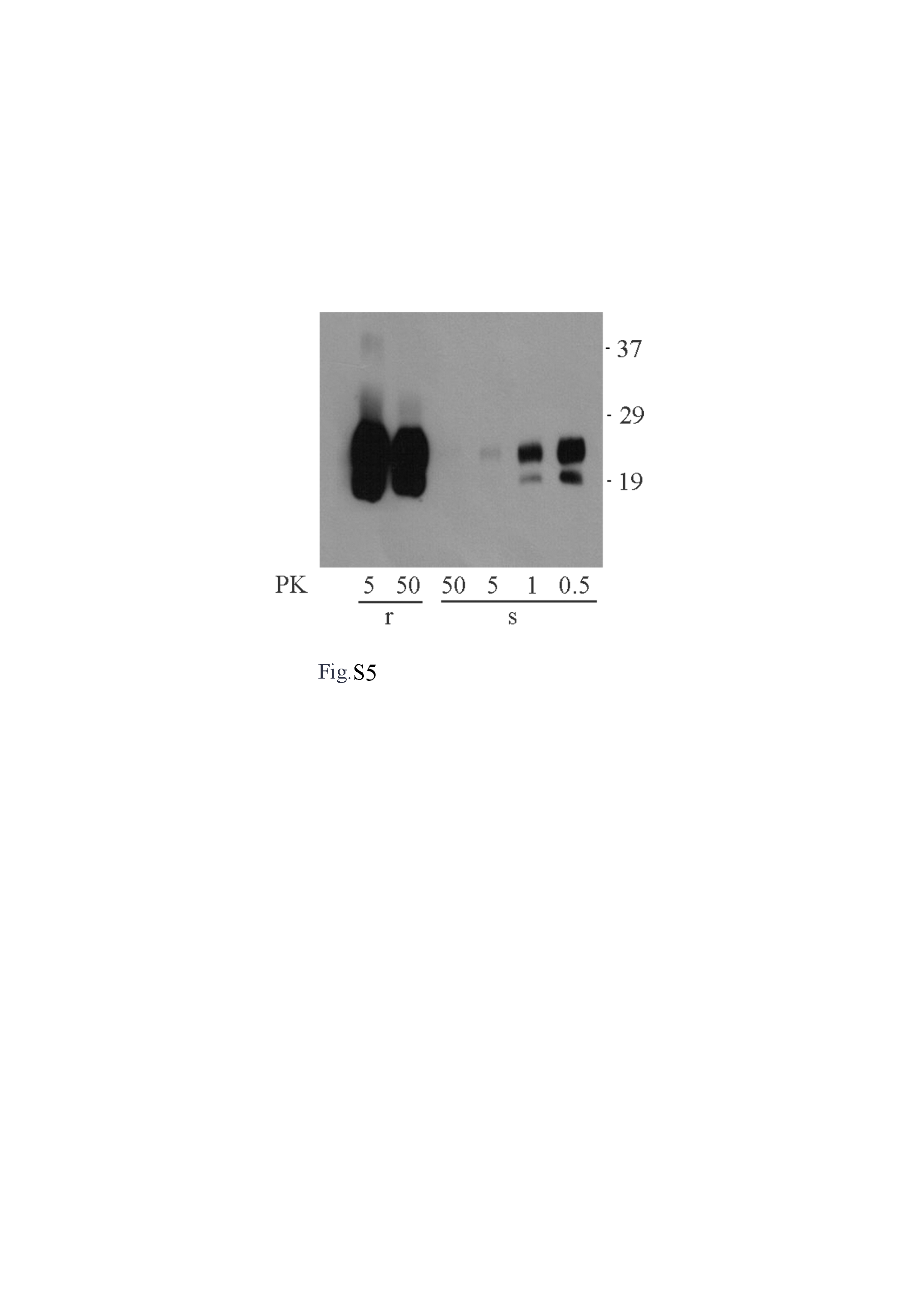

Supplement: Figure S5 — Comparison of the PK resistance of Dy rPrPSc and sPrPSc. Dy PrPSc fractions, rPrPSc (r) and sPrPSc (s), were isolated (see Materials and Methods) and treated with the indicated concentrations of PK and analyzed by WB (probed with mAb 3F4). (TIF) [file ppat.1002547.s005.tif]

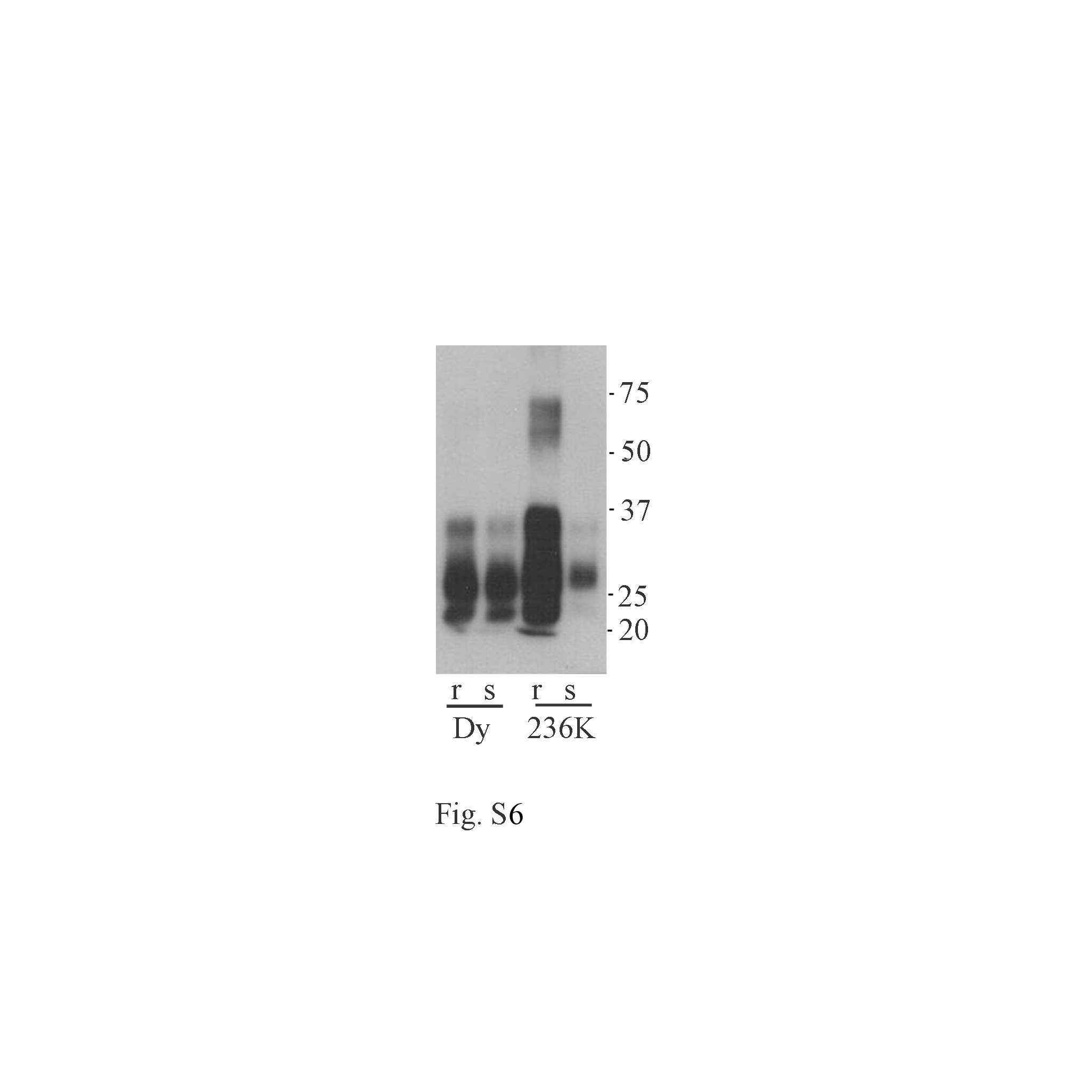

Supplement: Figure S6 — Relative amounts of sPrPSc in 263K and Dy PrPSc. Samples were probed with mAb 3F4. (TIF) [file ppat.1002547.s006.tif]

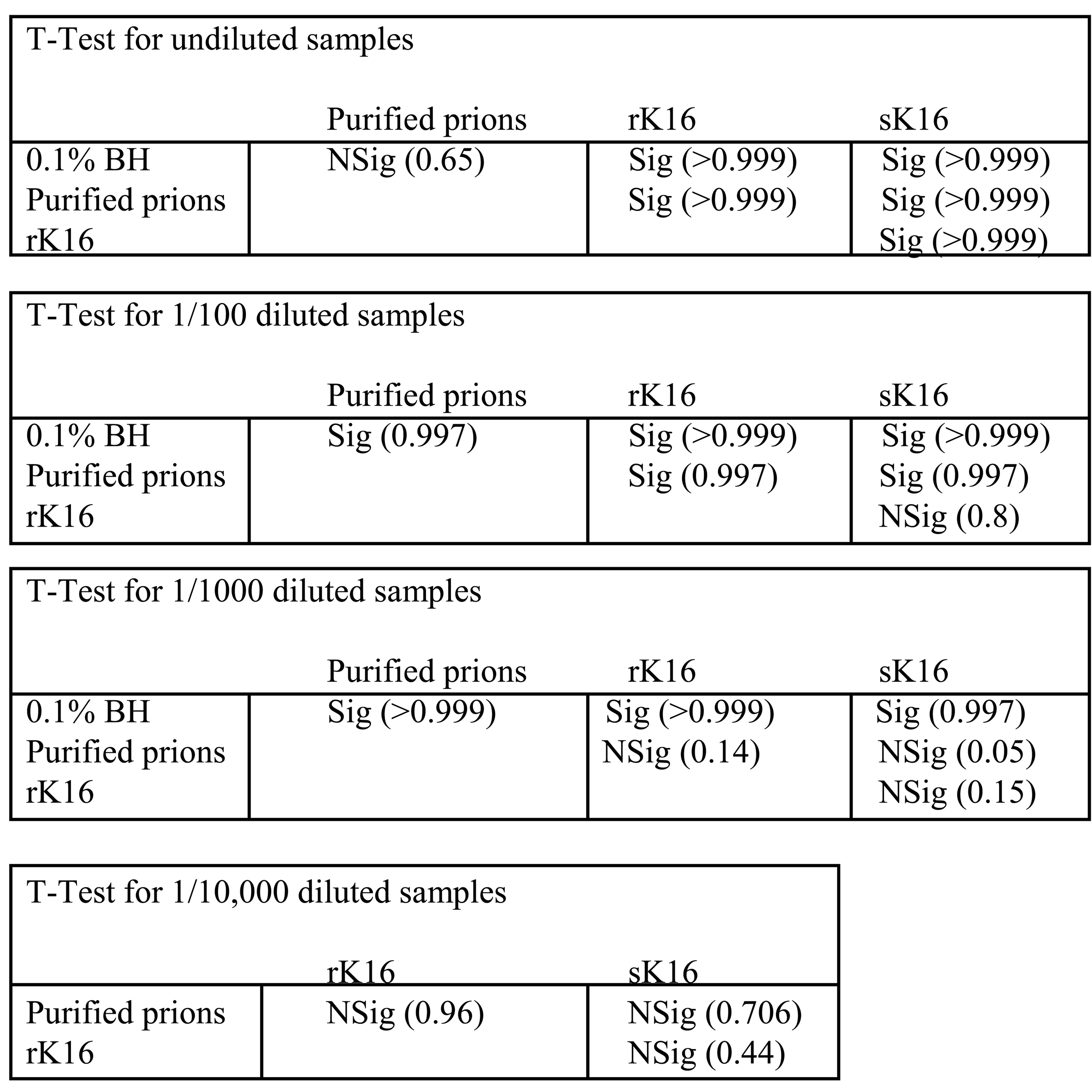

Supplement: Table S2 — Table of P-values for t-tests. The t-test was used to compare the Kaplan-Meier estimate data for the unpurified prions (0.1% BH), purified prions, PK resistant prions (rPrPSc), and the PK-sensitive prions (sPrPSc). The listed p-value is the result of a comparison of the Kaplan-Meier data for a row with that of a column. Each of the four dilutions (10−0, 10−2, 10−3, and 10−4) is in a separate table. A statistically significant difference (Sig) means that the P-value (in parentheses) is greater than or equal to 0.99. A statistically insignificant difference (NSig) means the P-value that is less than 0.99. (TIF) [file ppat.1002547.s008.tif]
