# Supplementary material for: PK-sensitive PrPSc Is Infectious and Shares Basic Structural Features with PK-resistant PrPSc
Source: PLoS Pathog. 2012 Mar 1;8(3):e1002547. doi: 10.1371/journal.ppat.1002547 (PMC3291653; doi:10.1371/journal.ppat.1002547)
Supplement: Table S1 — Table of Kaplan-Meier endpoint data. Tabular summary of the Kaplan-Meier estimate graphs for dilutions of four prion preparations (263K hamster-adapted scrapie). The unpurified PrPSc-containing 0.1% brain homogenate (Unpurified brain homogenate); the purified prions from a brain homogenate (purified prions); the PK sensitive prion fraction (sPrPSc); and the PK resistant prion fraction (rPrPSc). The data represents the time from inoculation to euthanization ± SD, as measured in days. Eight animals were used per dilution. Some dilutions were not done (n.d.). Only seven of the eight animals got sick after inoculation with the 10−4 dilution of sPrPSc fraction; the eighth remained healthy. (DOC) [file ppat.1002547.s007.doc]

| Supplementary Table S1 | | | | | |
| --- | --- | --- | --- | --- | --- |
| Survival times (days *pi*) of hamsters (n=8) inoculated with different prion inocula | | | | | |
| Inoculum | Dilution | | | | |
|  | **10^-0^** | **10^-1^** | **10^-2^** | **10^-3^** | **10^-4^** |
| Unpurified brain homogenate | 79±0 | 90±0 | 99±3 | 115±5 | n.d. |
| **Purified prions** | 79±1 | n.d. | 94±3 | 99±4 | 112±3 |
| **sPrP^Sc^** | 72±0 | n.d. | 87±4 | 98±4 | 110±6 |
| **rPrP^Sc^** | 75±2 | n.d. | 90±0 | 98±3 | 108±3 |
